# Dynamics and Complexity of a New 4D Chaotic Laser System

**DOI:** 10.3390/e21010034

**Published:** 2019-01-07

**Authors:** Hayder Natiq, Mohamad Rushdan Md Said, Nadia M. G. Al-Saidi, Adem Kilicman

**Affiliations:** 1 Institute for Mathematical Research, Universiti Putra Malaysia, UPM Serdang 43000, Malaysia; 2 The Branch of Applied Mathematics, Applied Science Department, University of Technology, Baghdad 10075, Iraq; 3 Malaysia-Italy Centre of Excellence for Mathematical Science, Universiti Putra Malaysia, UPM Serdang 43000, Malaysia; 4 Department of Mathematics, Universiti Putra Malaysia, UPM Serdang 43000, Malaysia

**Keywords:** Hopf bifurcation, self-excited attractors, multistability, sample entropy, PRNG

## Abstract

Derived from Lorenz-Haken equations, this paper presents a new 4D chaotic laser system with three equilibria and only two quadratic nonlinearities. Dynamics analysis, including stability of symmetric equilibria and the existence of coexisting multiple Hopf bifurcations on these equilibria, are investigated, and the complex coexisting behaviors of two and three attractors of stable point and chaotic are numerically revealed. Moreover, a conducted research on the complexity of the laser system reveals that the complexity of the system time series can locate and determine the parameters and initial values that show coexisting attractors. To investigate how much a chaotic system with multistability behavior is suitable for cryptographic applications, we generate a pseudo-random number generator (PRNG) based on the complexity results of the laser system. The randomness test results show that the generated PRNG from the multistability regions fail to pass most of the statistical tests.

## 1. Inroduction

The chaotic behavior as a rich nonlinear phenomenon has been detected in many non-natural and natural systems, and usually plays an important role in their performance [1,2]. Chaotic systems are complicated and have many interesting features, such as unpredictability, topological mixing, and high sensitivity to their initial conditions and parameters [3,4]. Therefore, chaotic systems have received significant attention from various fields including cryptography [5,6], secure communications [7,8], laser applications [9,10], biomedical engineering [11,12], and many others.

Existing chaotic systems can be classified into two categories: systems with self-excited attractors and systems with hidden attractors [13]. The chaotic system with self-excited attractors has a basin of attraction that is intersected with an unstable equilibrium, whereas the chaotic system with hidden attractors has a basin of attraction which does not intersect with any open neighborhoods of equilibria [14,15]. According to the above definition, most of the classical chaotic attractors are self-excited [16,17]. Meanwhile, it has been demonstrated that the attractors in dynamical systems with no equilibria [18,19], stable equilibria [20], lines of equilibria [21], and curves of equilibria [22] are hidden attractors.

However, with further investigation of chaos, it was unexpected to find that many systems with self-excited and hidden attractors have more than one attractor for a given set of parameters and different initial values. This phenomenon is known as multistability or coexisting attractors. The clear evidence of multistability was first experimentally manifested in a Q-switched gas laser [23], since then chaotic systems with multistability behaviors have been extensively reported. Munoz et al. presented a fractional-order chaotic system with multiple coexisting attractors [24]. Wang et al. established a 2D chaotic map with no-equilibria generating a pair of chaotic attractors [25]. Li et al. introduced a new method for constructing self-reproducing chaotic systems with extreme multistability [26]. In fact, multistability as a new research direction in chaos theory requires further research, especially, how to determine and locate this complicated nonlinear phenomenon in the chaotic systems.

Since the in-depth analysis of the local bifurcation is required to clarify the evolution of the chaotic state from the steady state, the scope of studying the bifurcation of the equilibria in the chaotic systems is of considerable interest [27]. Hopf bifurcation is one of an important local dynamic bifurcation, and is considered as the emergence of a limit cycle from an equilibrium point. Furthermore, the Hopf bifurcation plays a crucial role in analyzing the stability of the equilibria of the high-dimensional system [28,29]. Therefore, Hopf bifurcation is beneficial to analyzing the dynamic behavior of high-dimensional chaotic systems, as well as to the applications of controlling chaos [30].

Complexity of nonlinear dynamical systems has attracted attention in recent years due to its importance for measuring the predictability and randomness of the system time series [31,32]. The time series with high complexity led to a chaotic attractor, hence, the complexity is able to determine and locate the chaotic and periodic attractors in nonlinear systems [33,34]. Motivated by this observation, this paper applies Sample Entropy contour plot to determine multistability regions of a new 4D chaotic laser system, which is derived from Lorenz-Haken equations. The new chaotic system has one unstable equilibrium and symmetric stable equilibria, hence the chaotic attractor of the presented system is generally self-excited, meanwhile, the possible existence of a hidden chaotic attractor is an open problem.

The main contributions of this research work are as follows:

(i) We derive a new 4D chaotic laser system with three equilibria from Lorenz-Haken equations;
(ii) We investigate the stability of the symmetric equilibria, and the existence of coexisting multiple Hopf bifurcations on these equilibria;
(iii) We analyze the presence of complex coexisting behaviors in the laser system;
(iv) We use the complexity of the laser system time series to locate the regions of coexisting attractors when the parameters and initial values vary;
(v) Based on the complexity of the system time series, we study the randomness of multistability regions.

The rest of this paper is organized as follows: Section 2 introduces the new 4D chaotic laser system and studies its dynamical properties. Section 3 investigates the existence of Hopf bifurcation in the laser system. Section 4 provides the details about the multistability of the laser system. In Section 5, we use SamEn to locate the regions of the coexisting attractors, as well as to demonstrate the randomness of these regions. The conclusions are presented in Section 6.

## 2. A New 4D Chaotic Laser System From Lorenz-Haken Model

In this section, we discuss the dynamics of a new 4D chaotic laser system which is derived from the well-known Lorenz-Haken equations [35]. In the standard notation of Reference [36], the Lorenz-Haken equations is given by

(1) $$\left\{\begin{array}{c} \begin{array}{cc} \frac{dx}{dt} & =-\sigma \left(x-y\right)+{iqx|x|}^{2},\\ \frac{dy}{dt} & =-(1-i\delta )y+(r-z)x,\\ \frac{dz}{dt} & =-bz+Re(x*y). \end{array} \end{array}\right.$$

In the optical language, *x* is proportional to the electric field, *y* is proportional to the induced macroscopic polarization, (r-z) denotes the inversion, $\sigma =\frac{\tau_{P}}{\tau_{E}}$, and $b=\frac{\tau_{P}}{\tau_{N}}$. Here, $\tau_{E}$ represents the optical field, $\tau_{P}$ is the induced polarization, and $\tau_{N}$ denotes the inversion parameter. Meanwhile, the parameter δ governs the coupling between amplitude and phase variations, and *q* is known as the linewidth enhancement factor.

Since both *x* and *z* can be chosen to be real [37], the dynamics of Equation (1) can be investigated by considering the following linear transformation

$$\begin{array}{c} x=x_{1},\;\;\;\;\;y=x_{2}+ix_{3},\;\;\;\;\;z=x_{4}. \end{array}$$

Consequently, the new 4D chaotic laser system is defined as

(2) $$\begin{array}{c} \left\{\begin{array}{c} \begin{array}{cc} \frac{dx_{1}}{dt} & =\sigma (x_{2}-x_{1}),\\ \frac{dx_{2}}{dt} & =-x_{2}-\delta x_{3}+\left(r-x_{4}\right)x_{1},\\ \frac{dx_{3}}{dt} & =\delta x_{2}-x_{3},\\ \frac{dx_{4}}{dt} & =-bx_{4}+x_{1}x_{2}, \end{array} \end{array}\right. \end{array}$$

where $x_{i}$ are state variables and σ,δ,r and *b* are parameters.

### 2.1. Chaotic Behavior Regions

**Figure 1:**
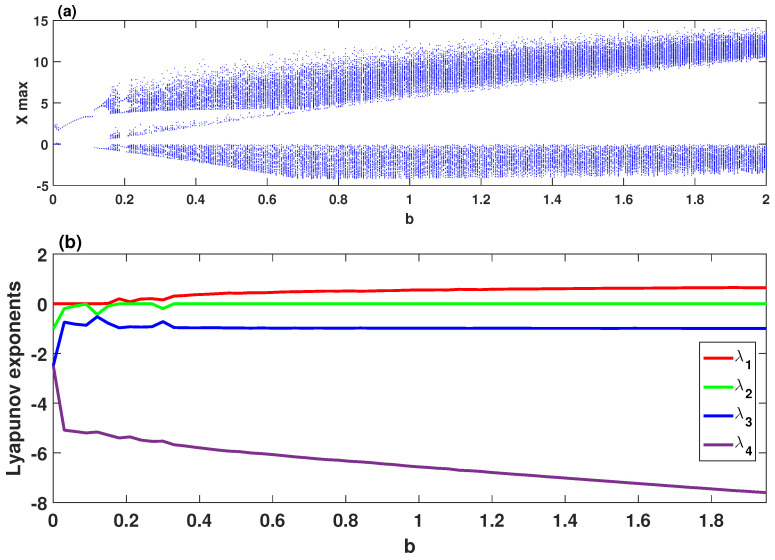
Dynamics of the system (2) versus the parameter *b* for the initial values (2,1,1,2) and with σ=4, δ=0.5, r=27: (**a**) bifurcation diagram; (**b**) Lyapunov exponents.

**Figure 2:**
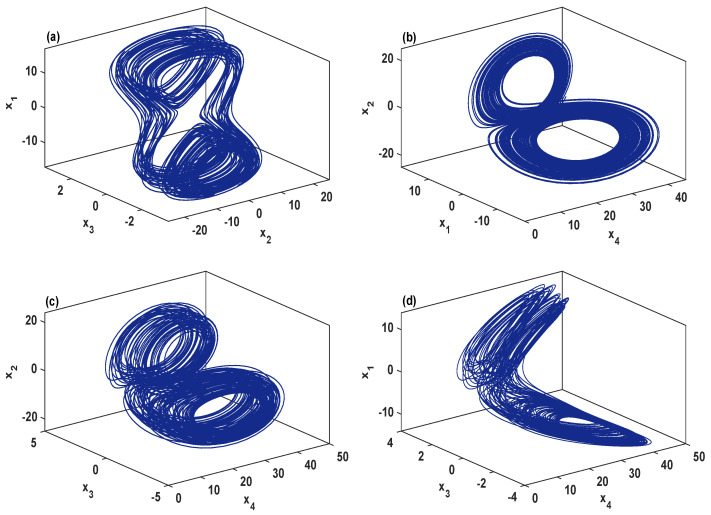
Different orientations on a two-scroll chaotic attractor of the system (2) for the initial values (2,1,1,2) and with the parameters σ=4, δ=0.5, r=27, b=2. (**a**) $\left(x_{2},x_{3},x_{1}\right)$ space; (**b**) $\left(x_{4},x_{1},x_{2}\right)$ space; (**c**) $\left(x_{4},x_{3},x_{2}\right)$ space; (**d**) $\left(x_{4},x_{3},x_{1}\right)$ space.

To examine the dynamic characteristics of the system (2), Figure 1a,b depicts its bifurcation diagram and Lyapunov exponents, respectively, in which the parameters are set as σ=4, δ=0.5, r=27, and 0≤b≤2. This figure clearly shows chaotic attractors for b∈[0.15,0.187]∪[0.205,2], quasi-periodic (when b=0.132) and periodic attractors for b∈[0,0.15)∪(0.187,0.205). To demonstrate the chaotic behavior of the system (2), Figure 2 plots its phase portraits with σ=4, δ=0.5, r=27, b=2 and for the initial values (2,1,1,2). As can be observed in Figure 2, the system (2) has a two-scroll chaotic attractor.

### 2.2. Dissipation and Symmetry

The divergence of system (2) is defined as

$$\begin{array}{c} \nabla V=\frac{\partial {\dot{x}}_{1}}{\partial x_{1}}+\frac{\partial {\dot{x}}_{2}}{\partial x_{2}}+\frac{\partial {\dot{x}}_{3}}{\partial x_{3}}+\frac{\partial {\dot{x}}_{4}}{\partial x_{4}}=-\left(\sigma +b+2\right). \end{array}$$

Thus, the system (2) becomes dissipative when (σ+b+2)>0. This means each volume element $V_{0}e^{-(\sigma +b+2)t}$ of system (2) shrinks to zero as t⟶∞ at an exponential rate (σ+b+2).

Additionally, the system (2) has invariance under the coordinate transformation

$$\begin{array}{c} \left(x_{1},x_{2},x_{3},x_{4}\right)⟶\left(-x_{1},-x_{2},-x_{3},x_{4}\right). \end{array}$$

Consequently, the system (2) has rotational symmetry around the $x_{4}$-axis.

### 2.3. Equilibria and Stability

Suppose that the parameters σ>0, δ>0, r>0 and b>0, then the equilibria of the system (2) can be calculated by solving the following equations

$$\begin{array}{c} \left\{\begin{array}{c} \begin{array}{c} \sigma (x_{2}-x_{1})=0,\\ -x_{2}-\delta x_{3}+\left(r-x_{4}\right)x_{1}=0,\\ \delta x_{2}-x_{3}=0,\\ -bx_{4}+x_{1}x_{2}=0. \end{array} \end{array}\right. \end{array}$$

From the above equations, it can be obtained that the equilibria of the system (2) have the following form:

$$\begin{array}{c} E_{i}\left(k,\;\;k,\;\;\delta k,\;\;\frac{k^{2}}{b}\right), \end{array}$$

where *k* is either 0 or $\pm \sqrt{b(r-\left(1+\delta^{2}\right))}$. The system (2) has one real equilibrium $E_{1}\left(0,0,0,0\right)$ when $r=1+\delta^{2}$, whereas it has three real equilibria if $r>1+\delta^{2}$

$$\begin{array}{c} \left\{\begin{array}{c} \begin{array}{cc} E_{1} & (0,0,0,0),\\ E_{2} & \left(\sqrt{b(r-\left(1+\delta^{2}\right))},\sqrt{b(r-\left(1+\delta^{2}\right))},\delta \sqrt{b(r-\left(1+\delta^{2}\right))},r-\left(1+\delta^{2}\right)\;\right),\\ E_{3} & \left(-\sqrt{b(r-\left(1+\delta^{2}\right))},-\sqrt{b(r-\left(1+\delta^{2}\right))},-\delta \sqrt{b(r-\left(1+\delta^{2}\right))},r-\left(1+\delta^{2}\right)\;\right). \end{array} \end{array}\right. \end{array}$$

Using the Jacobian matrix, the system (2) is linearized at the equilibrium $E_{i}$ as follows

$$\begin{array}{c} \begin{array}{c} J_{E_{i}}=\left(\begin{array}{cccc} -\sigma & \sigma & 0 & 0\\ r-\frac{k^{2}}{b} & -1 & -\delta & -k\\ 0 & \delta & -1 & 0\\ k & k & 0 & -b \end{array}\right). \end{array} \end{array}$$

Since the equilibria $E_{2,3}$ are symmetric about the $x_{4}-$axis, then they will have the same characteristics. Therefore, the characteristic equation of Jacobian matrix at the equilibrium $E_{2}$ with b=1 can be written as

(3) $$\begin{array}{c} f\left(\lambda \right)=\left(\lambda +1\right)f_{1}\left(\lambda \right)=0, \end{array}$$

where

(4) $$\begin{array}{c} f_{1}\left(\lambda \right)=\lambda^{3}+\left(2+\sigma \right)\lambda^{2}+\left(1+2\sigma +\delta^{2}+k^{2}+\sigma k^{2}-\sigma r\right)\lambda +\left(\sigma +\sigma \delta^{2}+3\sigma k^{2}-\sigma r\right) \end{array}.$$

It is obvious that Equation (3) always has one eigenvalue with negative real part which is $\lambda_{1}=-1$, whereas the real parts of the other eigenvalues are not always negative. It is well-known that a system is asymptotically stable when all eigenvalues have negative real parts; otherwise, the system is unstable. By Routh–Hurwitz criterion, the real parts of all the eigenvalues of the system (2) are negative if and only if

$$\begin{array}{c} \left\{\begin{array}{c} \begin{array}{cc} \Delta_{1}= & (2+\sigma )>0,\\ \Delta_{2}= & \left|\begin{array}{cc} \left(2+\sigma \right) & 1\\ \left(\sigma +\sigma \delta^{2}+3\sigma k^{2}-\sigma r\right) & \left(1+2\sigma +\delta^{2}+k^{2}+\sigma k^{2}-\sigma r\right) \end{array}\right|>0,\\ \Delta_{3}= & \left(\sigma +\sigma \delta^{2}+3\sigma k^{2}-\sigma r\right)\Delta_{2}>0. \end{array} \end{array}\right. \end{array}$$

By choosing the parameters σ>0 and δ>0, these inequalities lead to the following condition:

(5) $$\begin{array}{c} r<\frac{4\sigma +\sigma^{2}-\sigma^{2}\delta^{2}}{\sigma -2} \end{array}.$$

Thus, if the above conditions are satisfied, then the equilibrium $E_{2}$ is an asymptotically stable.

## 3. Local Bifurcation Analysis and Numerical Simulations

This section reviews the Hopf bifurcation using the bifurcation theories. In addition, the existence of coexisting symmetric Hopf bifurcations in the system (2) will be investigated with the variation of parameter $r\in R^{+}$.

### 3.1. Hopf Bifurcation

Hopf bifurcation is the source of a limit cycle, which usually appears when the stability of the equilibrium point changes at some critical parameter value. To illustrate the Hopf bifurcation of a dynamical system on the equilibrium point, consider a vector field as follows

(6) $$\begin{array}{c} \begin{array}{c} \dot{x}=f\left(x,\zeta \right), \end{array} \end{array}$$

where $x\in R^{4}$ and $\zeta \in R^{+}$ represent the phase variables and the parameters, respectively. The vector field undergoes a Hopf bifurcation when the following conditions are satisfied simultaneously [38]:

(A) nondegeneracy condition: the Jacobian matrix $J_{\left(x_{0},\zeta_{0}\right)}$ has one pair of purely imaginary roots, and other roots have nonzero real parts;
(B) transversality condition: the real part of differentiation characteristic equation with respect to the parameter ζ satisfy
  (7) $$\begin{array}{c} \begin{array}{c} Re\left(\frac{d\lambda }{d\zeta }\right){|}_{\zeta =\zeta_{0}}\neq 0; \end{array} \end{array}$$
(C) the first Lyapunov coefficient $l_{1}$ is nonzero.

In order to derive the first Lyapunov coefficient $l_{1}$, suppose that Equation (2) has an equilibrium point at $x=x_{0}$. By denoting $X=x-x_{0}$, we can write

(8) $$\begin{array}{c} \begin{array}{c} F\left(X\right)=f\left(X,\zeta_{0}\right), \end{array} \end{array}$$

as

(9) $$\begin{array}{c} \begin{array}{c} F\left(X\right)=AX+\frac{1}{2}B\left(X,X\right)+\frac{1}{6}C\left(X,X,X\right)+O\left(∥X{∥}^{4}\right), \end{array} \end{array}$$

where *A* is the Jacobian matrix, and *B* and *C* are symmetric multilinear vector functions which are defined as

(10) Bi(X,Y)=∑j,k=1n∂2Fi(η)∂ηj∂ηk|η=0XjYk,i=1,2,⋯,n,Ci(X,Y,Z)=∑j,k,l=1n∂3Fi(η)∂ηj∂ηk∂ηl|η=0XjYkZl,i=1,2,⋯,n.

Suppose that *A* possesses a pair of purely imaginary eigenvalues $\lambda_{1,2}=\pm i\omega$, meanwhile, the other eigenvalues have nonzero real part. Let p,q be an eigenvectors of *A* satisfying the following three conditions

(11) $$\begin{array}{c} \left\{\begin{array}{c} \begin{array}{c} Aq=i\omega_{0}q,\\ A^{T}p=-i\omega_{0}p,\\ \langle p,q\rangle =\sum_{i=1}^{n}\bar{p_{i}}\;q_{i}=1. \end{array} \end{array}\right. \end{array}$$

By means of an immersion of the form $X=V(\mu ,\bar{\mu })$, the 2D center manifold associated to the eigenvalues $\lambda_{1,2}=\pm i\omega$ is parameterized, where $V:C^{2}⟶R^{4}$ has a Taylor expansion of the following form

(12) $$\begin{array}{c} \begin{array}{c} V\left(\mu ,\bar{\mu }\right)=\mu \;q+\bar{\mu }\;\bar{q}+\sum_{2\leq j+k\leq 3}\frac{1}{j!k!}v_{jk}\;\mu^{j}\;{\bar{\mu }}^{k}+{O(|\mu |}^{4}). \end{array} \end{array}$$

with $v_{jk}\in C^{4}$ and $v_{jk}={\bar{v}}_{jk}$. By substituting Equation (12) into (8), one has

(13) $$\begin{array}{c} \begin{array}{c} \frac{\partial V}{\partial \mu }\dot{\mu }+\frac{\partial V}{\partial \bar{\mu }}\dot{\bar{\mu }}=F\left(V\left(\mu ,\bar{\mu }\right)\right) \end{array} \end{array}$$

Defined by the coefficients $\mu^{j}\;{\bar{\mu }}^{k}$, the complex vectors $v_{jk}$ can be obtained by solving Equation (13). On the chart μ for a center manifold, the system (13) can be written as

(14) $$\begin{array}{c} \begin{array}{c} \dot{\mu }=i\omega_{0}\mu +\frac{1}{2}G_{21}{\mu |\mu |}^{2}+{O(|\mu |}^{4}). \end{array} \end{array}$$

Thus, the first Lyapunov coefficient can be defined as

(15) $$\begin{array}{c} \begin{array}{c} l_{1}=\frac{1}{2\omega_{0}}Re\left[\langle p,C\left(q,q,\bar{q}\right)\rangle -2\langle p,B\left(q,-v_{11}\right)\rangle +\langle p,B\left(\bar{q},v_{20}\right)\rangle \right] \end{array} \end{array}$$

where $v_{11}=-A^{-1}B\left(q,\bar{q}\right)$ and $v_{20}={\left(2i\omega_{0}I-A\right)}^{-1}B\left(q,q\right)$.

### 3.2. Numerical Simulations

To investigate the existence of Hopf bifurcation in the system (2) at the equilibrium $E_{2}$, we will examine the conditions (A), (B) and (C) one by one.

Firstly, we assume that the characteristic Equation (3) has a pair of purely imaginary eigenvalues $\lambda_{1,2}=\pm i\omega_{0}$. By substituting $\lambda =i\omega_{0}$ into (4), one has

(16) -iω03-(2+σ)ω02+(1+2σ+δ2+k2+σk2-σr)iω0+(σ+σδ2+3σk2-σr)=0,

which leads to:

$$\begin{array}{c} \left\{\begin{array}{c} \begin{array}{c} -i\omega_{0}^{3}+\left(1+2\sigma +\delta^{2}+k^{2}+\sigma k^{2}-\sigma r\right)i\omega_{0}=0,\\ -\left(2+\sigma \right)\omega_{0}^{2}+\left(\sigma +\sigma \delta^{2}+3\sigma k^{2}-\sigma r\right)=0. \end{array} \end{array}\right. \end{array}$$

Thus, one can obtain that

$$\begin{array}{c} \left\{\begin{array}{c} \begin{array}{c} \omega_{0}=\sqrt{\frac{\sigma +\sigma \delta^{2}+3\sigma k^{2}-\sigma r}{2+\sigma }},\\ r=\frac{2+4\sigma +2\delta^{2}+2k^{2}+2\sigma^{2}+\sigma^{2}k^{2}}{\sigma +\sigma^{2}}, \end{array} \end{array}\right. \end{array}$$

which are equivalent to

$$\begin{array}{c} \left\{\begin{array}{c} \begin{array}{c} \omega_{0}=\sqrt{\frac{2\sigma k^{2}}{2+\sigma }},\\ r=\frac{4\sigma +\sigma^{2}-\sigma^{2}\delta^{2}}{\sigma -2}, \end{array} \end{array}\right. \end{array}$$

where $k=\sqrt{r-(1+\delta^{2})}$. It is worth noting that when $r=r_{0}=\frac{2+4\sigma +2\delta^{2}+2k^{2}+2\sigma^{2}+\sigma^{2}k^{2}}{\sigma +\sigma^{2}}$, the characteristic Equation (3) can be written as

(17) $$\begin{array}{c} f\left(\lambda \right)=\left(\lambda +1\right)\left(\lambda +2+\sigma \right)\left(\lambda^{2}+\frac{\sigma +\sigma \delta^{2}+3\sigma k^{2}-\sigma r_{0}}{2+\sigma }\right) \end{array}.$$

Therefore, the four eigenvalues of the system (2) are as follows

(18) $$\begin{array}{c} \left\{\begin{array}{c} \begin{array}{c} \lambda_{1}=-1,\\ \lambda_{2}=-\left(2+\sigma \right),\\ \lambda_{3}=i\sqrt{\frac{\sigma +\sigma \delta^{2}+3\sigma k^{2}-\sigma r_{0}}{2+\sigma }}=i\omega_{0},\\ \lambda_{4}=-i\sqrt{\frac{\sigma +\sigma \delta^{2}+3\sigma k^{2}-\sigma r_{0}}{2+\sigma }}=-i\omega_{0}. \end{array} \end{array}\right. \end{array}$$

Consequently, the nondegeneracy condition (A) is satisfied when $r=r_{0}$.

Secondly, let $\lambda \left(r\right)=\pm i\omega_{0}\left(r\right)$, by substituting λ(r) into Equation (10) and differentiate the both sides with respect to *r*, one obtains

(19) $$\begin{array}{c} \begin{array}{c} \frac{d\lambda (r)}{dr}=\frac{\sigma \lambda +\sigma }{3\lambda^{2}+2\left(2+\sigma \right)\lambda +\left(1+2\sigma +\delta^{2}+k^{2}+\sigma k^{2}-\sigma r\right)}, \end{array} \end{array}$$

which leads to:

(20) $$\begin{array}{c} \begin{array}{c} \frac{d\lambda (r)}{dr}{|}_{r=r_{0},\lambda =i\omega_{0}}=\frac{\sigma (i\omega_{0})+\sigma }{3{\left(i\omega_{0}\right)}^{2}+2\left(2+\sigma \right)i\omega_{0}+\left(1+2\sigma +\delta^{2}+k^{2}+\sigma k^{2}-\sigma r_{0}\right)}, \end{array} \end{array}$$

Thus, one has

(21) $$\begin{array}{c} \begin{array}{c} Re\left(\lambda^{\prime }\left(r=r_{0}\right)\right)=\frac{\sigma (1+2\sigma +\delta^{2}+k^{2}+\sigma k^{2}+\omega_{0}^{2}+2\sigma \omega_{0}^{2}-\sigma r_{0})}{{\left(1+2\sigma +\delta^{2}+k^{2}+\sigma k^{2}-\sigma r-3\omega_{0}^{2}\right)}^{2}+4{\left(2+\sigma \right)}^{2}\omega_{0}^{2}}>0, \end{array} \end{array}$$

where σ=4, δ=1.1, $r_{0}=\frac{4\sigma +\sigma^{2}-\sigma^{2}\delta^{2}}{\sigma -2}\approx 6.32$ and $\omega_{0}=\sqrt{\frac{2\sigma k^{2}}{2+\sigma }}\approx 2.34$. Consequently, the transversality condition (B) is also verified.

At last, we will calculate the first Lyapunov coefficient $l_{1}$ under the above fixed parameters. The Jacobian matrix *J* on the equilibrium point $E_{2}$ is given by

(22) $$\begin{array}{c} \begin{array}{cc} J_{E_{2}}= & \left(\begin{array}{cccc} -4 & 4 & 0 & 0\\ 2.21 & -1 & -1.1 & -2.0273\\ 0 & 1.1 & -1 & 0\\ 2.0273 & 2.0273 & 0 & -1 \end{array}\right). \end{array} \end{array}$$

The proper eigenvectors *q* and *p* are obtained by straightforward calculations

(23) $$\begin{array}{c} \begin{array}{c} q=\left(\begin{array}{c} 0.274+0.333i\\ 0.079+0.494i\\ 0.21+0.052i\\ 0.717 \end{array}\right),\;\;\;\;\;\;\;p=\frac{1}{\left(0.078-0.891i\right)}\left(\begin{array}{c} -0.318+0.073i\\ -0.7\\ 0.118+0.278i\\ 0.219+0.512i \end{array}\right) \end{array} \end{array}$$

where the above eigenvectors *q* and *p* satisfy the three conditions (11), namely

Aq=iω0q,ATp=-iω0p,〈p,q〉=∑i=1npi¯qi=1.

From Equation (10), the multilinear vector functions of the system (2) are calculated as follows

(24) $$\begin{array}{c} \begin{array}{c} B\left(x,y\right)=\left(\begin{array}{c} 0\\ -x_{1}y_{4}-x_{4}y_{1}\\ 0\\ x_{1}y_{2}+x_{2}y_{1} \end{array}\right),\;\;\;\;\;C\left(x,y,z\right)=\left(\begin{array}{c} 0\\ 0\\ 0\\ 0 \end{array}\right), \end{array} \end{array}$$

From (22)–(24), it follows that

$$\begin{array}{c} \begin{array}{c} B\left(q,q\right)=\left(\begin{array}{c} 0\\ -0.393-0.477i\\ 0\\ -0.285+0.323i \end{array}\right),\;\;\;\;\;\;\;B\left(q,\bar{q}\right)=\left(\begin{array}{c} 0\\ -0.393\\ 0\\ 0.372 \end{array}\right), \end{array} \end{array}$$

$$\begin{array}{c} \left\{\begin{array}{c} \begin{array}{c} J_{E_{2}}^{-1}=\left(\begin{array}{cccc} -0.192 & -0.121 & 0.133 & 0.246\\ 0.057 & -0.121 & 0.133 & 0.246\\ 0.063 & -0.133 & -0.852 & 0.271\\ -0.272 & -0.493 & 0.542 & 0 \end{array}\right),\\ {\left(2i\omega_{0}I-J_{E_{2}}\right)}^{-1}=\left(\begin{array}{cccc} 0.052-0.135i & -0.117-0.098i & 0.027-0.021i & 0.051-0.039i\\ -0.038-0.074i & -0.002-0.235i & 0.053+0.01i & 0.097+0.019i\\ -0.018+0.005i & -0.053-0.01i & 0.048-0.215i & 0.009-0.021i\\ -0.085-0.024i & -0.148+0.019i & 0.002-0.034i & 0.048-0.267i \end{array}\right). \end{array} \end{array}\right. \end{array}$$

Thus, one obtains

(25) $$\begin{array}{c} \left\{\begin{array}{c} \begin{array}{c} v_{11}={\left[-0.139,\;-0.139,\;-0.153,\;-0.193\right]}^{T},\\ v_{20}={\left[-0.002+0.122i,\;-0.145+0.119i,\;0.019+0.038i,\;0.14+0.155i\right]}^{T}. \end{array} \end{array}\right. \end{array}$$

By using (23)–(25), one gets

(26) $$\left\{\begin{array}{c} \begin{array}{c} \langle p,B\left(q,-v_{11}\right)\rangle =0.066-0.191i,\\ \langle p,B\left(\bar{q},v_{20}\right)\rangle =0.064-0.130i,\\ \langle p,C\left(q,q,\bar{q}\right)\rangle =0. \end{array} \end{array}\right.$$

Consequently, the first Lyapunov is obtained by substituting (26) into (15)

$$\begin{array}{c} \begin{array}{c} l_{1}=\frac{1}{2\omega_{0}}Re\left[\langle p,C\left(q,q,\bar{q}\right)\rangle -2\langle p,B\left(q,-v_{11}\right)\rangle +\langle p,B\left(\bar{q},v_{20}\right)\rangle \right]=-0.0145<0. \end{array} \end{array}$$

**Figure 3:**
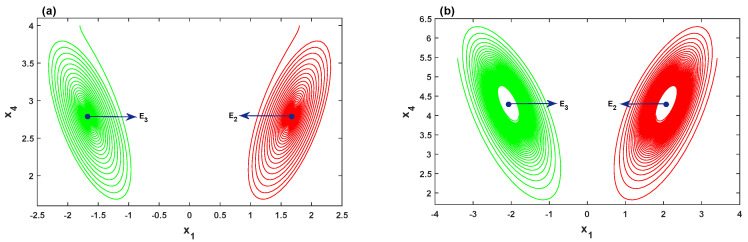
Hopf bifurcation of the system (2): (**a**) $r=5.5<r_{0}$, the orbit of the system is attracted to the stable symmetric equilibria $E_{2}$ and $E_{3}$; (**b**) $r=6.5>r_{0}$, the orbit of the system is attracted to a stable limit cycle emerging from the symmetric equilibria $E_{2}$ and $E_{3}$.

Therefore, the Hopf bifurcation of the system (2) at equilibrium point $E_{2}$ is nondegenerate and supercritical. Furthermore, the equilibria $E_{2}$ and $E_{3}$ are symmetric about the $x_{4}-$axis, hence, the system (2) should also undergo a Hopf bifurcation at $E_{3}$. Two numerical simulations are given in Figure 3. For $r=5.5<r_{0}$, the orbit of the system (2) with the initial values (1.8,1.8,2,4) is attracted to the stable equilibrium point $E_{2}$, whereas the orbit with the initial values (-1.8,-1.8,-2,4) is attracted to the other stable equilibrium point $E_{3}$, as illustrated in Figure 3a. In Figure 3b, by choosing $r=6.5>r_{0}$ with the initial values (1.8,1.8,2,4) and (-1.8,-1.8,-2,4), the orbits of the system are attracted to stable limit cycles emerging from $E_{2}$ and $E_{3}$, respectively.

According to Reference [39], m=2, τ=1 and r=0.1∼0.2 times standard deviation (SD) of the time series. In our experiment, we fix m=2, τ=1 and r=0.2×SD.

## 4. Multistability Behavior

A nonlinear dynamical system with multistability behavior can generate two or more attractors simultaneously depending on the initial values of the system. This section investigates the existence of multistability behavior in the system (2).

**Figure 4:**
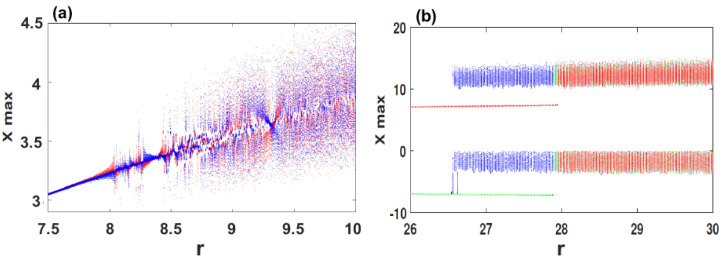
Bifurcation diagrams versus parameter *r* for illustrating the two and three coexisting attractors of the system (2): (**a**) σ=2, δ=1.5, b=0.7 for the initial values (1,1,1,1) (red) and (-2,1,1,1) (blue); (**b**) σ=4, δ=0.5, b=2 for the initial values (2,1,1,2) (blue), (-2,1,1,-2) (red) and (2,1,1,-2) (green).

**Figure 5:**
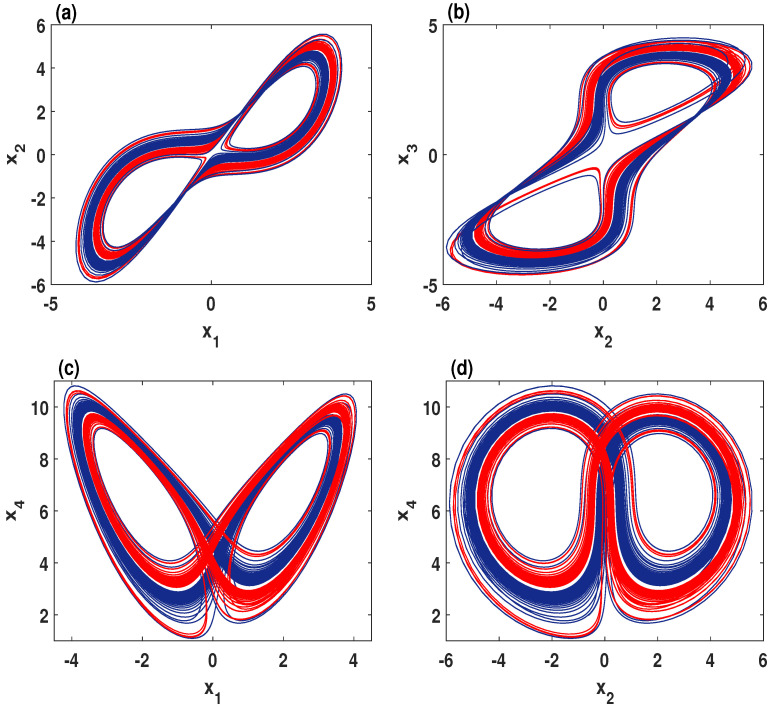
Multiple coexisting chaotic attractors of the system (2) when σ=2, δ=1.5, b=0.7, r=9.41 for the initial values (1,1,1,1) (red) and (-2,1,1,1) (blue). (**a**) $x_{1}$–$x_{2}$ plane; (**b**) $x_{2}$–$x_{3}$ plane; (**c**) $x_{1}$–$x_{4}$ plane; (**d**) $x_{2}$–$x_{4}$ plane.

When we fix the parameters σ=2, δ=1.5, b=0.7 and select *r* as bifurcation parameter for over the range r∈[7.5,10], the coexisting bifurcation models of the state variable $x_{1}$ are depicted in Figure 4a. In this figure, the attractor colored in blue is initiated from (-2,1,1,1), meanwhile the attractor colored in red begins with the initial conditions (1,1,1,1). As can be observed in Figure 4a, the system (2) shows coexisting multiple chaotic attractors as well as the coexistence of multiple quasi-periodic attractors. To show the coexistence of multiple chaotic attractors visually, Figure 5 plots different orientations of the phase portraits of the system (2) when its parameters are set as σ=2, δ=1.5, b=0.7, and r=9.41.

**Figure 6:**
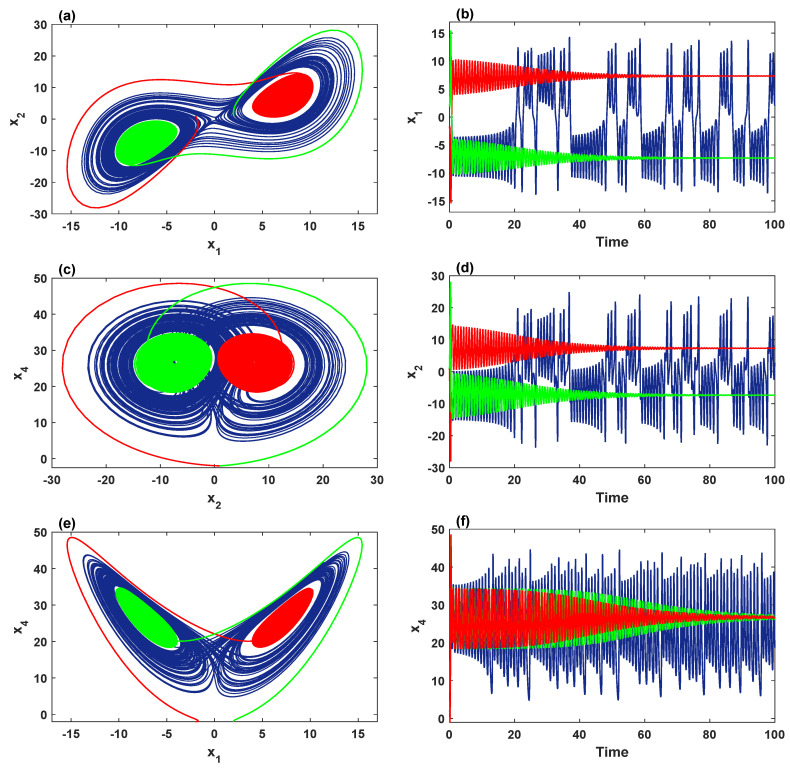
Three coexisting attractors with σ=4, δ=0.5, b=2, r=27: (**a**,**c**,**e**) different perspectives on the coexistence of the chaotic and two stable fixed-point attractors for the initial values (2,1,1,2) (blue), (-2,1,1,-2) (red) and (2,1,1,-2) (green); (**b**,**d**,**f**) the corresponding time series of the state variables $x_{1}$, $x_{2}$ and $x_{4}$, respectively.

In addition, when we set σ=4, δ=0.5, b=2 with 26≤r≤30, Figure 4b shows that the chaotic attractor with two stable fixed-point attractors coexist for the initial values (±2,1,1,±2). For the orbit colored in blue, the evolution begins from attracting to the stable equilibrium $E_{3}$ within the range 26≤r≤26.7, and then the system shows chaotic behavior when r≥26.8. For (-2,1,1,-2) (red), the system converges to the stable equilibrium $E_{2}$ when 26≤r≤28, and then exhibits chaotic behavior when r≥28.1. For the initial values (2,1,1,-2) (green), the system attracts to the stable equilibrium $E_{3}$ when 26≤r≤27.8, meanwhile the chaotic behavior is shown when r≥27.9. Selecting r=27, an interesting dynamic is observed in the system (2) by plotting different orientations of the phase portraits with the corresponding time series, as shown in Figure 6. These portraits confirm the coexistence of three different attractors: (a) blue butterfly attractors surrounds the symmetric equilibria $E_{2}$ and $E_{3}$; (b) the red stable fixed-point attractor for $E_{2}$, and the green stable fixed-point attractor for $E_{3}$.

Through the above analysis, we can observe that the multistability behavior occurs in the system (2) with various kinds of coexisting attractors. Therefore, it can be concluded that the system (2) has high sensitivity to both initial values and parameters.

## 5. Complexity and Randomness of Multistability Regions

This section discusses determining and locating the parameters and initial values that show multistability behaviors, as well as investigates the randomness of the multistability regions.

### 5.1. Sample Entropy

Sample Entropy (SamEn) is a mathematical algorithm proposed by Richman [40]. It is used to provide a quantitative explanation about the complexity of nonlinear dynamical systems. Obviously, a system with bigger SamEn values indicates that it requires additional information to predict its attractor, hence, it is a chaotic system. Suppose that the time series $\left(y_{i},i=0,1,2,\ldots ,M-1\right)$ of a dynamical system with a length of *M*, then the SamEn algorithm can be calculated by the following steps:

(A) Reconstructing phase-space: for a given embedding dimension *m* and time delay τ, the reconstruction sequences are given by
  (27) $$\begin{array}{c} \begin{array}{c} Y_{i}=\left\{y_{i},y_{i+\tau },...,y_{i+(m-1)\tau }\right\},\;\;\;y_{i}\in R^{m} \end{array} \end{array}$$
  where i=1,2,⋯,M-m+τ.
(B) Counting the vector pairs: let $B_{i}$ be the number of vector $Y_{j}$ such that
  (28) $$\begin{array}{c} \begin{array}{c} d[Y_{i},Y_{j}]\leq r,\;\;\;i\neq j \end{array} \end{array}$$
  where *r* is the tolerance parameter, and $d[Y_{i},Y_{j}]$ is the distance between $Y_{i}$ and $Y_{j}$, which is defined by
  (29) $$\begin{array}{c} \begin{array}{c} d\left[Y_{i},Y_{j}\right]=max\{|y\left(i+k\right)-y\left(j+k\right)\left|:0\leq k\leq m-1\right\}. \end{array} \end{array}$$
(C) Calculating probability: according to the obtained number of vector pairs, we can obtain
  (30) $$\begin{array}{c} \begin{array}{cc} C_{i}^{m}\left(r\right) & =\frac{B_{i}}{M-(m-1)\tau }, \end{array} \end{array}$$
  then calculate the probability by
  (31) $$\begin{array}{c} \begin{array}{cc} \phi^{m}\left(r\right) & =\frac{\sum_{i=1}^{M-(m-1)\tau }lnC_{i}^{m}\left(r\right)}{\left[M-(m-1)\tau \right]} \end{array} \end{array}$$
(D) Calculating SamEn: repeating the above steps we can obtain $\phi^{m+1}\left(r\right)$, then SamEn is given by
  (32) $$\begin{array}{c} \begin{array}{c} SamEn\left(m,r,M\right)=\phi^{m}\left(r\right)-\phi^{m+1}\left(r\right). \end{array} \end{array}$$

According to Reference [39], m=2, τ=1 and r=0.1∼0.2 times standard deviation (SD) of the time series. In our experiment, we fix m=2, τ=1 and r=0.2×SD.

It is well-known that the cross-section of the basins of attraction can determine the dynamical system behaviors when its initial values vary. However, it is interesting to ask if there is any technique that can determine the behaviors of a dynamical system when its initial values and parameters vary. Therefore, SamEn based contour plots are applied to locate the regions of chaotic and periodic state, and hence, to determine the parameters and initial values that show multistability behaviors. To locate those parameters and initial values in the system (2), we designed the following experiments:  (1) consider *r* as bifurcation parameter and set σ=4, b=2 and δ=0.5; (2) let $\left(x_{10},x_{20},x_{30},x_{40}\right)$ be the initial values; (3) calculate SamEn versus varying the parameter *r* and one of an initial value; (4) calculate SamEn versus varying two of the initial values.

**Figure 7:**
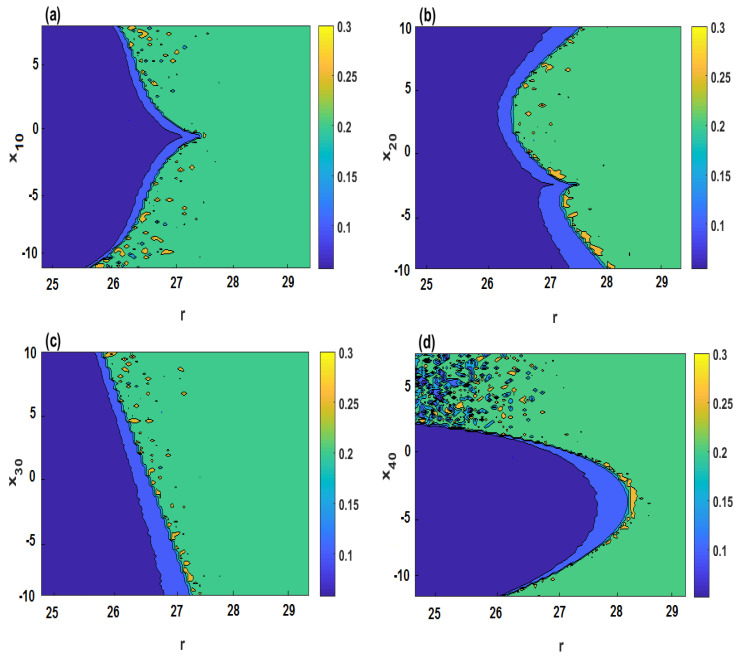
SamEn in the parameter *r*-initial value plane for σ=4, δ=0.5, b=2: (**a**) $r-x_{10}$ plane; (**b**) $r-x_{20}$ plane; (**c**) $r-x_{30}$ plane; (**d**) $r-x_{40}$ plane.

**Figure 8:**
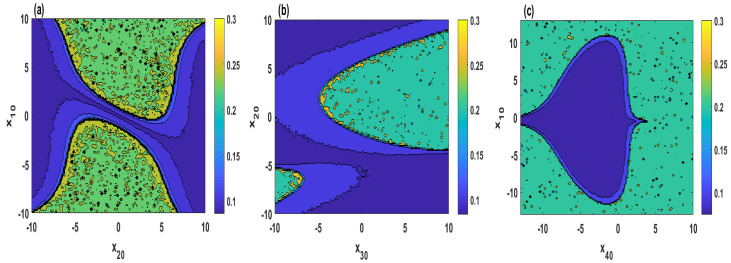
SamEn versus varying two of the initial values for σ=4, δ=0.5, b=2, r=27: (**a**) $\left(x_{10},x_{20},1,2\right)$; (**b**) $\left(2,x_{20},x_{30},2\right)$; (**c**) $\left(x_{10},1,1,x_{40}\right)$.

Figure 7 plots SamEn of the system (2) in a two-dimensional plane when r∈(24,30) and different initial values. It can be observed from Figure 7a–d that four cases are analyzed when the initial values are set as $\left(x_{10},1,1,2\right)$, $\left(2,x_{20},1,2\right)$, $\left(2,1,x_{30},2\right)$ and $\left(2,1,1,x_{40}\right)$, respectively. From Figure 7, it can be seen that the parameter *r* and the initial values in the blue regions have smaller SamEn values, which means that the system (2) shows periodic state, whereas, those in the yellow and green regions lead to a chaotic state. Furthermore, Figure 8 shows the chaotic and periodic regions of system (2) when two of the initial values vary simultaneously.

### 5.2. Chaos-Based PRNG

Many chaotic systems have been applied to generate pseudorandom number generator (PRNG). The need of PRNG arises in many cryptographic applications, e.g., common cryptosystems employ keys, data hiding, and auxiliary quantities used in generating digital signatures [41,42]. However, secret keys of most chaos-based cryptographic schemes are generated by parameters and initial values of the employed chaotic systems [43]. Those parameters and initial values might be from multistability regions; it is therefore important to investigate the randomness of the trajectories generating from multistability regions.

To investigate the randomness of blue-green regions (multistability behaviors) and green regions (chaotic), which is shown in Figure 7d, we use here a simple chaos-based PRNG as an example. The generation procedures of the chaos-based PRNG are shown in Algorithm 1, for which $x_{1}$, $x_{2}$, $x_{3}$ and $x_{4}$ generates 1,000,000 bits binary string.

Several statistical tests can be employed to test the randomness of PRNG. Our experiment uses the highest standards of statistical packages which is NIST-800-22 [42]. The NIST-800-22 consists of 16 empirical statistical tests that provide true evaluation for the randomness of PRNG. Each test is developed to detect the non-random areas of a binary sequence from different sides, and then to derive a *p*-value. According to the recommendations in [24,44], we set the confidence level α=0.01, and we use a binary sequence with length of 1,000,000 bit as the testing input. Since the confidence level of each test in NIST is set to be 1%, then the sequence is considered to be random with a confidence of 99% when the obtained *p*-value is bigger than 0.01.

**Figure 9:**
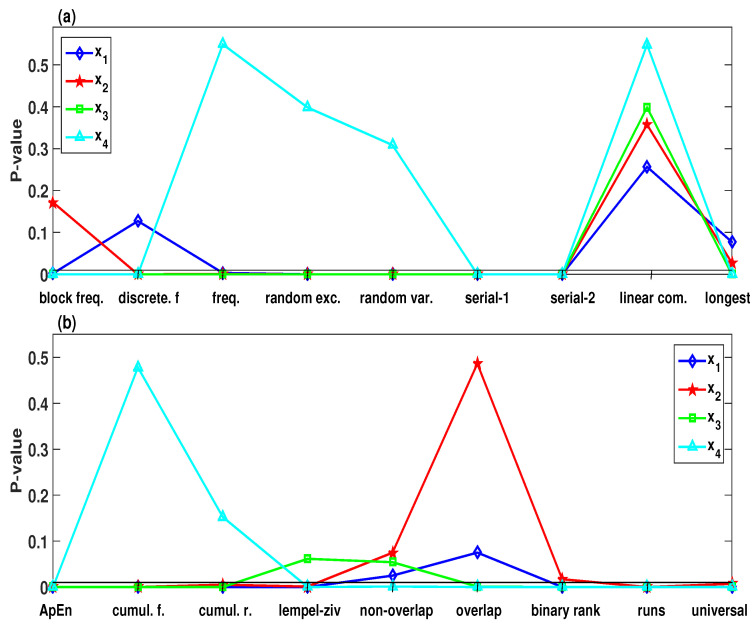
The statistical tests NIST SP800-22 of the pseudorandom number generator (PRNG) that generated by $x_{1}$, $x_{2}$, $x_{3}$, $x_{4}$ of the system (2) with σ=4, δ=0.5, b=2, r∈[27,29] and for the initial values (2,1,1,-2). (**a**) Block-Frequency, Discrete Fourier Transform, Frequency (Monobit), Random Excursions, Random Excursions Variant, Serial-1, Serial-2, Linear Complexity, and Longest Run of Ones, respectively; (**b**) Approximate Entropy, Cumulative Sums (Forward), Cumulative Sums (Reverse), Lempel-ziv Compression, Non-overlapping Template, Overlapping Template, Binary Matrix Rank, Runs, and Universal Statistical.

According to Algorithm 1, we can obtain four PRNG from the trajectory of $x_{1}$, $x_{2}$, $x_{3}$ and $x_{4}$ when the initial values are considered as input. For σ=4, δ=0.5, b=2 and r∈[27,29] with the initial values (2,1,1,-2), the SamEn values of the selected parameters and initial values are within the blue-green regions (multistability), as shown in Figure 7d. The randomness of the corresponding PRNG that generated from the trajectory of $x_{1}$, $x_{2}$, $x_{3}$ and $x_{4}$ can be visually shown by depicting the NIST-800-22 test results, as seen in Figure 9. As can be observed from Figure 9, the four PRNG generating from multistability regions fail to pass most of the statistical tests. On the other hand, when σ=4, δ=0.5, b=2 and r∈[27,29] with the initial values (2,1,1,2), the SamEn values are within the green region (chaotic), as shown in Figure 7d. Table 1 lists the corresponding NIST-800-22 results for each of the four PRNG. It is obvious that the four PRNG can pass all the statistical tests.

**Algorithm 1** The generation of chaos-based PRNG**Input:** The initial values of system (2).
1:**for**i=1 to 4 **do**2:    **for** r=27 to 29 **do**3:        Truncate a chaotic sequence $C_{i}$ from the trajectory of $x_{i}$;4:        Convert the floating number $C_{i}$ of $x_{i}$ into a 32-bit binary using the IEEE-754-Standard;5:        Fetch the last 16th digital number of the obtained binary string;6:    **end for**7:**end** **for**
**Output:** Four PRNG are generated from of $x_{1}$, $x_{2}$, $x_{3}$ and $x_{4}$


## 6. Conclusions

This paper has introduced a new 4D chaotic laser system, which is derived from Lorenz-Haken equations. The new chaotic laser system has three equilibria and only two quadratic nonlinearities. The dynamics of the new system have been studied deeply, in which the system shows coexisting multiple Hopf bifurcations, and complex coexisting behaviors of two and three attractors. In addition, we applied SamEn contour plots for measuring the complexity of the system when its initial values and parameters vary. Simulation results have shown that multistability regions can be easily determined and located using SamEn contour plots. To examine the randomness of PRNG that generate from the multistability regions, we used the NIST-800-22 tests. Statistical test results demonstrate that the generated PRNG from multistability regions are non-random. This means that although the multistability behaviors indicate high sensitivity of chaotic systems, they might be unsuitable for cryptographic applications.

## Figures and Tables

**Table 1:** NIST-800-22 tests results of binary sequences generated by PRNG of $x_{1}$, $x_{2}$, $x_{3}$ and $x_{4}$ outputs.

Each Sequence to be Tested Consists of 1,000,000 Bits						
**NIST-800-22 Tests**		p **-Value (** $x_{1}$ **)**	p **-Value (** $x_{2}$ **)**	p**-Value** ($x_{3}$**)**	p **-Value (** $x_{4}$ **)**	**Result**
1.	Block-Frequency (m = 128)	0.2116	0.8460	0.8313	0.0210	Random
2.	Frequency (Monobit)	0.7611	0.0380	0.6570	0.3503	Random
3.	Discrete Fourier Transform	0.3602	0.1792	0.1478	0.1225	Random
4.	Approximate Entropy (m = 10)	0.9592	0.6512	0.6343	0.3659	Random
5.	Cumulative Sums (Forward)	0.7617	0.0721	0.7280	0.5832	Random
	Cumulative Sums (Reverse)	0.5578	0.0320	0.5106	0.1816	Random
6.	Serial-1 (m = 16)	0.7937	0.2948	0.1635	0.9706	Random
	Serial-2 (m = 16)	0.8885	0.7628	0.5357	0.9530	Random
7.	Runs	0.9649	0.6196	0.4751	0.1530	Random
8.	Longest Run of Ones	0.2568	0.0965	0.8242	0.2420	Random
9.	Overlapping Template (m = 9)	0.7032	0.6461	0.5603	0.7085	Random
10.	Non-overlapping Template (m = 9)	0.4960	0.5403	0.5150	0.5117	Random
11.	Linear Complexity (m = 500)	0.4091	0.7263	0.1607	0.8582	Random
12.	Binary Matrix Rank	0.2618	0.1029	0.2843	0.2376	Random
13.	Lempel-ziv Compression	0.0769	0.2343	0.1411	0.9581	Random
14.	Random Excursions	0.4628	0.2379	0.4787	0.3931	Random
15.	Random Excursions Variant	0.6141	0.1814	0.3977	0.2865	Random
16.	Universal Statistical	0.4931	0.7326	0.6056	0.1038	Random
